# Editorial: New trends in natural product research for inflammatory and infectious diseases: Volume II

**DOI:** 10.3389/fphar.2023.1144074

**Published:** 2023-01-26

**Authors:** Jaime Ribeiro-Filho, Yanna Carolina Ferreira Teles, John Ogbaji Igoli, Raffaele Capasso

**Affiliations:** ^1^ Oswaldo Cruz Foundation (FIOCRUZ), Fiocruz Ceará, Eusébio, Brazil; ^2^ Department of Chemistry and Physics, Federal University of Paraíba, JoãoPessoa, Brazil; ^3^ Department of Chemical Sciences, Pen Resource University, Gombe, Nigeria; ^4^ Department of Agricultural Sciences, University of Naples Federico II, Portici, Italy

**Keywords:** natural products, inflammation, infection, drug development, preclinical research, pharmacology

## 1 Introduction

Natural compounds and extracts have been a consistent source in the search for new drugs to treat several diseases, including inflammations and infections. One of the pharmaceutical industry’s most successful compounds is the anti-inflammatory drug acetylsalicylic acid, developed from an active compound of the plant species *Salix alba* (Fijałkowski et al., 2022). Colchicine from *Colchicum autumnale* is considered one of the oldest remedies in use today as an anti-inflammatory drug against gout and other diseases (Dasgeb et al., 2018).

In the last decade, the relevance of research on drug discovery from natural sources was acknowledged by the Physiology/Medicine Nobel Prizes awards in 2015. The prize was awarded to the researchers working on novel natural therapeutical antiparasitic drugs, avermectin and artemisinin, used against global infectious diseases mainly occurring in neglected communities from developing countries (Tambo et al., 2015).

Along with the acknowledgment of natural products research comes the challenge of identifying and quantifying the highly variable composition of natural extracts, selecting the lead compounds from a large number of promising compounds, as well as understanding their biochemical action and mechanism. The modern instrumental techniques for identifying and quantifying compounds, and the chemoinformatic, with its several experimental tools, have enabled medicinal chemists to achieve fast and effective drug discovery programs that have yielded promising results (Cox and Gupta, 2022). The most remarkable example of the combined use of modern techniques was the research to identify the molecular mechanism of the SARS-CoV-2 virus and the search to develop new treatments for COVID-19 and its complications (Mtewa et al., 2022).

Diseases of inflammatory and infectious origin represent significant challenges to global health, becoming among the most relevant fields in pharmaceutical and medical research. Notably, the currently available medicines have significant therapeutic limitations and long-term use toxicity concerns that point to the need to pursue new anti-inflammatory and anti-infectious drugs (Miranda et al., 2021).

In this second volume of the Research Topic, we obtained manuscripts focusing on fully characterized natural products, using updated spectroscopic techniques to demonstrate the potential of natural extracts and compounds against inflammatory and infectious diseases.

## 2 Discussion

In the first published manuscript of this Research Topic, Liu et al. demonstrated that brevilin A, a sesquiterpene lactone isolated from the Chinese species *Centipeda minima*, dose-dependently inhibited the production of nitric oxide (NO) and decreased the PGE_2_ secretion by LPS/IFNγ-stimulated murine macrophages. Brevilin A was also shown to effectively decrease the expression of iNOS and COX-2 and markedly diminish the production of pro-inflammatory cytokines. In the *in vivo* model of intratracheal instillation of LPS, brevilin A significantly suppressed the infiltration of alveolar macrophages, neutrophils, and epithelial cells in the bronchoalveolar lavage fluid. In addition, the treatment with the compound substantially ameliorated pathological parameters such as edema, hyperemia, alveolar collapse, and leukocyte infiltration. The authors also showed that the anti-inflammatory activity demonstrated by brevilin A is significantly related to its α, β-unsaturated ketone moiety. The researchers proposed the chemical mechanism of interaction of brevilin A and their most likely protein targets using *in silico* tools. They showed that the α, β-unsaturated ketone moiety of brevilin A has a favorable interaction with the cysteine (Cys114) residue on the active site of the human protein IKKβ *via* a covalent bond. This upstream kinase has a critical role in the NF-κB signaling pathway. Since NF-κB is a critical transcriptional factor in inflammatory events, the inhibition of its signaling pathway by brevilin A could explain the potent anti-inflammatory effects observed both *in vitro* and *in vivo*.

In the second manuscript, Xavier et al. focused on schistosomiasis, a highly prevalent neglected tropical disease. The manuscript reports the preparation and characterization of carvacrol nanoemulsion and the effects of its oral administration on *Schistosoma mansoni*-infected mice. Carvacrol, a monoterpene identified in essential oils extracted from many plant species, especially oregano (*Origanum vulgare*), is known for its antimicrobial activity. The authors showed that carvacrol nanoemulsion reduced the worm burden and egg production more effectively than the unconjugated compound. The prepared nano-formulation remarkably reduced the infection rate and egg production compared with the standard drug praziquantel. The findings showed that carvacrol-loaded nanoemulsion improved anti-schistosome activity by reducing worm and egg burden. As such, it is a promising delivery system in the context of schistosomiasis treatment.

In the search for new drugs to treat COVID-19, the third manuscript of this Research Topic reports the effects of the herbal product Virofree against SARS-CoV-2 infection (Doan et al.). A library of drugs and herbal medicines was applied to a transcriptome-based screening to select candidates with the potential to inhibit COVID-19 molecular targets. From this screening, the herbal medicine virofree was selected. This natural product has well-established antioxidant and anti-inflammatory effects, especially quercetin, hesperidin, genistein, daidzein, and resveratrol. In silico analysis showed the potential of this natural product in inhibiting COVID-19 gene targets and acute respiratory distress syndrome (ARDS). Biochemical analysis showed that Virofree impaired the binding of different spikes to ACE2, especially Delta and Omicron. Virofree was found to increase miR-148b-5p levels, inhibit Mpro (SARS-CoV-2 protease), reduce LPS-induced TNF-α release, prevent cellular iron accumulation, and reduce *in vitro* the expression of proteins related to pulmonary fibrosis. Importantly, this pioneering study provides relevant data that may guide research targeting natural products for drug development research in the COVID-19 context.

Dias et al. conducted a systematic review of terpenes as bacterial efflux pump inhibitors in the last manuscript on this Research Topic. Considering that previous research demonstrated that terpenes could play a role in antibiotic resistance, the authors conducted a relevant systematic review of manuscripts investigating these natural products as bacterial efflux pump inhibitors. Using the Preferred Reporting Items for Systematic Reviews and Meta-Analyses (PRISMA) protocol, the authors selected 41 manuscripts reporting a total of 75 different terpenes, 63 bacterial strains, and 22 different efflux pumps. Frequency analysis identified carvacrol (a monoterpene) as the most frequently reported terpenes, while *Staphylococcus aureus* SA-1199B and NorA were the most frequently investigated bacterial strain and efflux pump (EP), respectively. The study demonstrated that terpenes could inhibit efflux pump-mediated resistance in Gram-positive and Gram-negative bacteria. However, the frequency of EP inhibition was higher in Gram-positive strains. Of note, this relevant work contributed to identifying compounds with the potential for developing new drugs to treat infections caused by antibiotic-resistant bacteria.

## 3 Concluding remarks

The Research Topic ‘*New trends in natural product research for inflammatory and infectious diseases: Volume II*’ presented relevant manuscripts using updated methods with findings focusing on the effectiveness of natural extracts and compounds against inflammatory and infectious diseases.
